# damidBind: an R/bioconductor package for differential DamID analysis and data exploration

**DOI:** 10.1093/bioinformatics/btag512

**Published:** 2026-07-13

**Authors:** Owen J Marshall

**Affiliations:** Menzies Institute for Medical Research, University of Tasmania, Hobart 7000, Australia

## Abstract

**Summary:**

DamID, and its cell-type specific adaptations, including Targeted DamID (TaDa) and Chromatin Accessibility TaDa (CATaDa), are now widely-adopted as techniques for the genome-wide profiling of DNA binding proteins. Despite this popularity, no dedicated software solution exists for identifying differentially bound or accessible loci, or differentially transcribed genes, between cell types using DamID. The R/Bioconductor package damidBind provides these functions, allowing an end-user to move from processed binding profiles to identifying differentially-bound loci in a reproducible, statistically appropriate and straightforward workflow.

**Availability and implementation:**

damidBind is an open-source R/Bioconductor package and freely available from Bioconductor at https://bioconductor.org/packages/damidBind/, and from GitHub at https://github.com/marshall-lab/damidBind. It is released under the GPLv3 licence.

## 1 Introduction

DamID-seq profiles genome-wide protein–DNA association by tethering a DNA adenine methylase (Dam) to a DNA-binding or chromatin-associated protein, producing local GATC methylation that can be detected by methylation-sensitive digestion and sequencing (van Steensel and Henikoff 2000, van Steensel *et al*. 2001, Marshall *et al*. 2016). Cell-type-specific adaptations such as TaDa (Southall *et al*. 2013, Marshall *et al*. 2016, Cheetham *et al*. 2018, Tosti *et al*. 2018) extend this approach to intact tissues without cell sorting or antibodies.

As well as profiling transcription factors and chromatin proteins, DamID-seq can be used to profile the occupancy of RNA polymerase over gene bodies (Southall *et al*. 2013). This readout provides a proxy for gene transcription, and has been successfully used for transcriptional profiling of different cell types (e.g. Southall *et al*. 2013, Marshall and Brand 2017, Doupé *et al*. 2018, Otsuki and Brand 2018, Gervais *et al*. 2019).

As Dam has a strong affinity for open chromatin, the DamID technique always obtains a Dam-only control profile alongside the Dam-fusion profile, and the final binding profile is a normalised log_2_ ratio of GATC methylation. The Dam-only profile can be used as a direct measure of cell-type-specific chromatin accessibility, providing a readout (Chromatin Accessibility TaDa, or CATaDa) similar to ATAC-seq (Aughey *et al*. 2018).

Multiple software tools exist for processing raw DamID-seq NGS reads to log_2_ ratio binding profiles and peak calls, including damidseq_pipeline (Marshall and Brand 2015), Daim (Tosti *et al*. 2018) and Damsel (Page *et al*. 2024). However, because DamID-seq produces log_2_ ratios over unevenly-spaced GATC fragments, standard ChIP-seq and RNA-seq differential analysis tools are poorly suited to these data.

The R/Bioconductor package damidBind addresses this gap, formalising the process of performing differential binding analysis on all forms of DamID-seq datasets. It applies the data curation and underlying statistical analysis appropriate for each DamID application, providing a reproducible, data-appropriate workflow from processed NGS data to biological insights.

## 2 Implementation

### 2.1 Architecture

damidBind is implemented as an R package within the Bioconductor (Gentleman *et al*. 2004) ecosystem. Genomic data are stored as GenomicRanges (Lawrence *et al*. 2013) objects, linked to genome annotations from AnnotationHub. Core statistical tests for differential analysis are handled by limma (Smyth 2005) and NOISeq (Tarazona *et al*. 2015), and results are returned as a DamIDResults S4 object containing results tables, together with input file and genome annotation metadata. Where applicable, parallel execution is implemented via BiocParallel (see Table S2, available as supplementary data at *Bioinformatics* online for benchmarking details).

### 2.2 Input data

The damidBind package assumes that the user has processed NGS files to bedGraph profile tracks. For differential protein binding or chromatin accessibility, the package also requires a predetermined set of peak calls for each condition, which can be provided as either external peak files (BED or GFF) or as a GenomicRanges R object.

**Figure 1:**
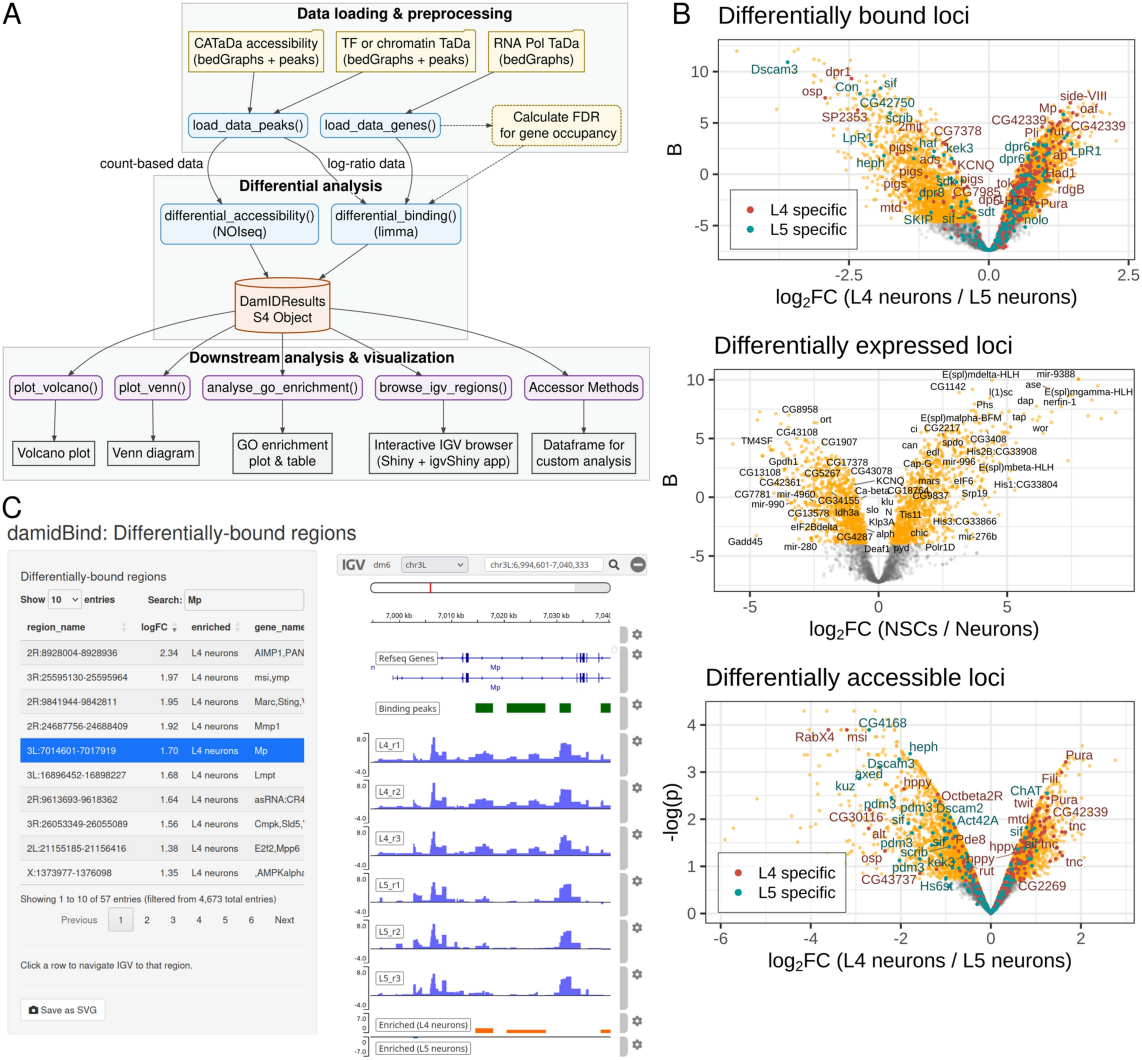
The damidBind package. (A) Schematic overview of the main functions and workflow of damidBind. (B) Example volcano plots of differential binding, expression and accessibility, illustrating different plot labelling options; data are from (Marshall and Brand 2017, 2015, Xu *et al*. 2024). (C) A screenshot of the Shiny/IGV based interface for interactive locus browsing.

A recommended workflow is NGS alignment and profile generation via damidseq_pipeline (Marshall and Brand 2015), and then peak-calling via either find_peaks (Marshall *et al*. 2016) (external, per replicate) or Damsel (Page *et al*. 2024) (within R, per condition) (Fig. S1; Table S1, available as supplementary data at *Bioinformatics* online), before commencing damidBind analysis (Fig. 1A).

At least three biological replicates per condition are recommended. Analyses with two replicates are supported, but have limited statistical power.

### 2.3 Data handling and normalisation

DamID-seq data are a distinct form of genomic binding/accessibility data which relies on methylated adenines in the sequence GATC. As a result, the underlying resolution of DamID data is the unevenly-spaced distribution of this sequence in the genome. This uneven GATC spacing makes standard count-based differential analysis tools inappropriate for DamID-seq.

At the core of damidBind’s analysis is a principle of maximising statistical power by reducing the number of statistical comparisons. For peaks-based analysis pipelines (CATaDa and conventional TaDa), per-replicate or per-condition peak calls are reduced to a set of unified binding regions. For expression analysis, gene body coordinates, as obtained from the most up-to-date genome release (or any historical release) via AnnotationHub, are used directly as binding regions.

The variable GATC fragment widths require spatial weighting to accurately represent locus occupancy. damidBind calculates the average, fragment-length-weighted signal for each replicate, $\sum_{i}si\mathrm{gna}l_{i}\times \mathrm{widt}h_{i}/\sum_{i}width_{i}$ for all fragments *i* within a locus, where *signal_i_* is either the fragment log_2_ enrichment for DamID binding data, or the reads-per-million count for CATaDa. This method correctly accounts for the variable fragment length structure of loci without being strongly influenced by length-based signal bias (see Supplementary Material  S.2.1; Fig. S2, available as supplementary data at *Bioinformatics* online). The locus-level weighted-means are used in all downstream analysis.

For all workflows, an option to apply uncentered scaling of the input data, along with quantile, cyclic LOESS or reads per million (RPM) normalisation is provided, prior to locus occupancy calculation (see Supplementary Material S.2.2, available as supplementary data at *Bioinformatics* online for further discussion of data normalisation strategies). Following data loading, damidBind provides diagnostic PCA and correlation heatmap plots of the input data, derived from both the underlying raw data and the mean locus occupancy values, allowing the user to check the data quality and condition separation.

### 2.4 Differential analysis workflows

#### 2.4.1 Protein binding

For conventional DamID or TaDa experiments, the damidBind workflow is initiated via load_data_peaks(). This function imports log_2_(Dam-fusion/Dam-only) binding profiles and associated peak calls, unifies peak regions across all replicates and calculates per-replicate peak occupancy scores. Peaks are also associated with genes in close proximity (within ± 1 kb of the gene body by default).

The subsequent analysis with differential_binding() uses the empirical Bayes (eBayes) framework of limma to identify statistically significant changes in protein binding between two conditions. limma is designed to handle heteroscedastic log_2_-ratio data with a mean-variance trend, as is typical in log_2_ ratio DamID binding data, and damidBind by default uses the trend and robust options of limma**’**s eBayes moderation to account for this (Fig. S3, available as supplementary data at *Bioinformatics* online).

#### 2.4.2 RNA polymerase occupancy

DamID can be used to profile RNA Polymerase occupancy across gene bodies as a proxy for gene expression. damidBind supports this via the load_data_genes() function, which calculates replicate occupancy over annotated genes. Differential analysis then proceeds using the same limma-based differential_binding() function as described above.

For RNA Polymerase datasets, damidBind provides the ability to determine the FDR of enriched gene body occupancy per condition, as a proxy for gene expression status. The algorithm used here is a substantially revised version of the approach described in Southall *et al*. (2013), introducing log-linear modelling with weighted natural splines and Jensen’s correction to model heteroscedasticity across fragment counts, and taking advantage of condition replicates to enrich statistical power (see Supplementary Material S.2.5, available as supplementary data at *Bioinformatics* online for algorithm details). The occupancy FDR values are not used directly in differential expression analysis, but can be used to limit downstream analysis and visualisation plots to expressed genes only.

#### 2.4.3 Chromatin accessibility

The Dam-only control sample from a DamID experiment can be used as a proxy for chromatin accessibility, via a method termed CATaDa (Aughey *et al*. 2018). Unlike log_2_ binding ratios, CATaDa data consist of count-derived, non-negative, non-normally-distributed accessibility signals, summarised as fragment-length-weighted averages over peaks. These data are under-dispersed locus-level weighted-average intensities rather than raw counts, and therefore do not naturally satisfy the raw-count assumptions of negative binomial workflows such as edgeR (Chen *et al*. 2025) or DESeq2 (Love *et al*. 2014) (Fig. S4, available as supplementary data at *Bioinformatics* online). The package’s differential_accessibility() function instead uses NOISeq (Tarazona *et al*. 2015) for differential analysis, a package designed to analyse normalised count-derived data non-parametrically, making it robust to this data type (see Supplementary Material S.2.3, Fig. S5 and Table S7, available as supplementary data at *Bioinformatics* online for further discussion and comparison with edgeR).

### 2.5 Scope and limitations

At the time of release, damidBind only handles pairwise comparisons between datasets. However, the underlying limma contrasts framework allows for more complex analyses, and these are planned for future releases. More complex CATaDa designs would require separate implementation and validation.

While damidBind is primarily designed to compare the same Dam-fusion protein across different contexts, the package can potentially be used to compare the binding of two different proteins in the same cell type (e.g. Nguyen *et al*. 2026). However, users should approach such analyses with caution, as different TFs may have markedly different binding profile distributions, making interpretation of differential binding and the assumptions of shared condition-level residual variance less secure.

### 2.6 Visualisation and downstream analysis

damidBind provides a number of tools for downstream functional analysis and publication-ready plots and figures. These include proportional Venn diagrams using biovenn (Hulsen *et al*. 2008), Gene Ontology enrichment analysis via clusterProfiler (Yu *et al*. 2012), customisable volcano plots (Fig. 1B; see also Supplementary Material S.2.4, available as supplementary data at *Bioinformatics* online), and a Shiny/igvShiny interface for browsing significant loci with replicate tracks, peaks and differential results (Fig. 1C). Because a core feature of damidBind is the association of bound loci to neighbouring genes, differential binding analysis is immediately linked to biological function in these outputs.

## 3 Application and validation

### 3.1 Differential binding and accessibility in lamina neurons

To assess the differential binding analysis of damidBind, the package was applied to a published TaDa dataset profiling the binding of the transcription factor Brain-specific homeobox (Bsh) in two related neuronal subtypes, L4 and L5 (Xu *et al*. 2024). Xu *et al*. (2024) used independent scRNA-seq data to identify marker genes uniquely transcribed in each neuronal subtype, finding that loci with enriched Bsh binding in Notch-ON L4 neurons were significantly associated with L4 specific markers. In contrast, in Notch-OFF L5 neurons, Bsh loci were depleted for L5 specific markers (Xu *et al*. 2024).

Compared against the analysis from (Xu *et al*. 2024), damidBind recovered almost all differentially bound loci identified in the earlier study (100% of L4 loci; 92% of L5 loci; Fig. S6A, Table S6, available as supplementary data at *Bioinformatics* online). The package also found substantial additional genes differentially bound by Bsh in both neuronal subtypes. Importantly, these additional targets showed the same association of loci with subtype-specific markers as the earlier study, with additional L4 loci enriched for L4-specific gene expression (odds ratio 2.33; $P<1.5\times 10^{-10}$), and L5 loci not linked with L5-specific gene expression (odds ratio 0.46; non-significant) (Fig. S6A, Table S4, available as supplementary data at *Bioinformatics* online). These data suggest that the damidBind reanalysis identified subtype-specific Bsh targets, consistent with increased sensitivity without obvious loss of biological specificity in this dataset.

A comparison between the original and damidBind analysis of the CATaDa samples in this paper was similar, with damidBind recovering 100% and 99% of all loci identified as significant in (Xu *et al*. 2024), and recovering additional subtype-specific expressed genes at a similar odds ratio (Fig. S6B, Table S8, Table S9, available as supplementary data at *Bioinformatics* online).

### 3.2 Differential transcription between neural stem cells and neurons

To assess the ability of damidBind to perform differential expression analysis, Targeted DamID RNA Polymerase II data for *Drosophila* larval neural stem cells and adult neurons was compared with an independent DESeq2 analysis of FACS-sorted bulk RNA-seq data from broadly corresponding cell types and developmental stages (data from Berger *et al*. 2012, Perlegos *et al*. 2024) (Fig. S7, available as supplementary data at *Bioinformatics* online).

As expected for an orthogonal comparison between polymerase occupancy and steady-state transcript abundance, concordance was substantial but incomplete, with a logFC correlation of 0.52 across all differentially-expressed genes in the datasets (Fig. S7A, B, available as supplementary data at *Bioinformatics* online), a directional concordance of 71% across all significant genes, and 80% for significant genes called by damidBind. When limited to genes with | log FC| > 2, this rose to >80% for both methods (Table S10, available as supplementary data at *Bioinformatics* online). Gene Ontology analysis showed that genes significant in both datasets were enriched for expected neural development/function categories, whereas RNA-seq-specific abundance changes were enriched for metabolic, splicing and ribosome biogenesis terms rather than neural development (Fig. S7C, available as supplementary data at *Bioinformatics* online). These differences may reflect post-transcriptional regulation, transcript half-life and driver/developmental-stage differences between the datasets.

Differential analysis of DamID-seq RNA Polymerase occupancy therefore recovers biologically meaningful, cell-identity-associated transcriptional differences, while remaining distinct from steady-state RNA abundance.

## 4 Conclusions

damidBind provides a standardised, comprehensive differential analysis package for DamID-seq data, encompassing all three major uses of the technique, with diagnostic plots and data visualisation functions that allow researchers to quickly identify key loci for downstream validation and further investigation. The package comes with a comprehensive vignette, and is freely available through the official Bioconductor software repository.

## Supplementary Material

btag512_Supplementary_Data

## Data Availability

No new primary sequencing data were generated for this study. The publicly available raw datasets analysed in this article were obtained from the NCBI Gene Expression Omnibus under accession numbers GSE247239, GSE77860, GSE69184, GSE235989, and GSE38764. Processed DamID-seq data and peak calls are available from Zenodo at https://doi.org/10.5281/zenodo.16649477. Data and code used to generate the main and supplementary analysis figures are available from Zenodo at https://doi.org/10.5281/zenodo.20758895 and from GitHub at https://github.com/marshall-lab/damidBind_manuscript_figures.

## Software availability

damidBind is available from Bioconductor at https://bioconductor.org/packages/damidBind/, with DOI 10.18129/B9.bioc.damidBind. The package source code is also available from GitHub at https://github.com/marshall-lab/damidBind. The archived package version used for the analyses in this manuscript, damidBind v1.1.1, is available from Zenodo at https://doi.org/10.5281/zenodo.20758869.
