# Supplementary material for: damidBind: an R/bioconductor package for differential DamID analysis and data exploration
Source: Bioinformatics. 2026 Jul 13;42(7):btag512. doi: 10.1093/bioinformatics/btag512 (PMC13391151; doi:10.1093/bioinformatics/btag512)
Supplement: btag512_Supplementary_Data [file btag512_supplementary_data.pdf]

Supplementary material

S.1 Supplementary methods

S.1.1 Data sources

Binding data (including CATaDa data) for Bsh (Xu *et al.*, 2024) was obtained from GEO accession number GSE247239. RNA Polymerase occupancy data (Marshall and Brand, 2017) was obtained from GEO accession numbers GSE77860 and GSE69184, processed with `damidseq_pipeline` v1.6.3 with default settings. Bulk RNA-seq data used in this study was obtained from GEO accession numbers GSE235989 (Adult 6D; Elav-GAL4 UAS-mCD8GFP control samples) (Perlegos *et al.*, 2024), and GSE38764 (third instar larval brain, wild-type neuroblasts) (Berger *et al.*, 2012).

The DamID-seq processed data and peak calls are provided in a Zenodo dataset at doi.org/10.5281/zenodo.16649477. This processed dataset consists of $\log_2$-ratio binding profiles, CATaDa RPM-normalised bedgraphs, alongside associated peak calls for the Bsh binding and CATaDa data.

S.1.2 DamID-seq data processing

DamID-seq data was processed with `damidseq_pipeline` v1.6.3 with either default settings (TF binding and RNA polymerase occupancy) or with the `--catata` flag (Dam-only samples for CATaDa). Peak calling was carried out using `find_peaks` v1.2 with default settings.

S.1.3 `damidBind` analysis

Differential binding / expression analysis was performed using `damidBind` v1.1.1 (Zenodo DOI: https://doi.org/10.5281/zenodo.20758869). Quantile normalisation was used as the default normalisation method unless otherwise specified; *Drosophila melanogaster* BDGP6.46 / Ensembl 113 (AH119285) was used as the genome annotation from `AnnotationHub`. All other settings used package defaults (including a $\pm$ 1kb window for gene associations with loci, q=0.8 as the NOISeq significance threshold for CATaDa analysis, and FDR<0.05 to filter for significant gene occupancy for RNA Polymerase II differential transcription analysis).

S.1.4 RNA-seq data processing and analysis

Raw FASTQ RNA-seq reads were aligned using `kallisto` v0.46.1, against the *D. melanogaster* BDGP 6.46 release 113, with `--genomebam -t 14` runtime flags. Transcript abundance `tsv` files were imported into R via `tximport`, analysed using `DESeq2`, and the results processed with `lfcShrink` using `apeglm` (Zhu *et al.*, 2019).

S.1.5 Additional analysis of CATaDa data

To assess the performance of `NOISeq` on CATaDa data, results were compared against the `edgeR` (v4.4.2) quasi-likelihood (QL) framework. Input data consisted of the same lamina neuron fragment-length-weighted RPM scores (summarised over peaks) used in the `damidBind` analysis. These scores were treated as fractional counts within `edgeR`; library size normalisation was performed using the trimmed mean of M-values (TMM) method via `calcNormFactors`, and common, trended, and tagwise dispersions were estimated using `estimateDisp`. Differential accessibility was determined using the `glmQLFit` and `glmQLFTest` functions, with significant loci defined at an FDR < 0.05.

Empirical null-call behaviour was evaluated using a permuted null test on all nine unique non-biological 3-versus-3 partitions (treating complementary partitions as equivalent and excluding the true L4-versus-L5 split, such that each group contained a mixture of biological conditions). Differential analysis was then performed on these pseudo-conditions using both `NOISeq` and `edgeR`.

S.1.6 Benchmarking

The main data processing steps for `damidBind` were benchmarked via the `bench` R package. Benchmarking was performed on both the RNA Polymerase II and Bsh TF binding datasets used in this study, using pre-loaded binding profile `GRanges` objects to minimise the contribution of disk I/O. The results are summarised in Table S2, for single core execution vs 15 and 30 cores. Benchmarks were performed on an AMD EPYC Milano server with 32 cores and 128 Gb RAM, running Ubuntu 22.04 and R 4.4.0.

S.1.7 Additional data analysis

All additional analysis was performed using R (R_Development_Core_Team, 2011) v4.4.0. Flowchart diagrams were generated using `DiagrammeR`. Composite plots were generated using `Patchwork`.

S.1.8 Figure analysis code

The code and data to generate all main and supplementary analysis figures is available at GitHub at https://github.com/marshall-lab/damidBind_manuscript_figures, with an archived snapshot at Zenodo, DOI: https://doi.org/10.5281/zenodo.20758895.

S.2 Supplementary methodological analyses

S.2.1 Effect of fragment-length-weighted averaging

In order to investigate the effects of fragment-length-weighted averaging of DamID-seq data, the relationship between $\log_2$ ratio signal and GATC fragment length was examined. The observed signal of any given fragment i can be considered as

$$S_i = B(L_i) + R_i,$$

where $B(L_i)$ is any systematic, fragment-length-dependent bias, and R_i is the residual signal, comprising true biological binding, stochastic technical noise, and any length-independent technical variation. For a genomic locus composed of multiple fragments, the signed discrepancy between the fragment-length-weighted mean and the simple mean is therefore

$$\Delta_{obs} = (\bar{B}_w - \bar{B}_s) + (\bar{R}_w - \bar{R}_s).$$

and the corresponding signed discrepancy expected from fragment-length bias alone is

$$\Delta_{bias} = \bar{B}_w - \bar{B}_s$$

Fragment-length bias was modelled for each sample by excluding all fragments overlapping a binding peak, which were considered to contain true signal for which $R_i \gg B(L_i)$. A generalised additive model was then fitted genome-wide to the remaining background fragments:

$$S_i = f(\log_{10}(L_i)) + \epsilon_i$$

where f was estimated using a cubic regression spline. Using this model, the expected length-associated signal $\hat{B}_i$ was predicted for all fragments within tested loci. Because the background set may still contain weak or undetected biological signal, this should provide a conservative upper estimate of fragment-length bias.

For each tested locus containing multiple fragments, the observed absolute discrepancy between the fragment-length-weighted mean and the simple mean was calculated as

$$D_{obs} = |\Delta_{obs}| = |\bar{S}_w - \bar{S}_s|$$

The corresponding absolute discrepancy expected from the fitted fragment-length bias model alone was calculated as

$$D_{bias} = |\Delta_{bias}| = |\bar{\hat{B}}_w - \bar{\hat{B}}_s|$$

These quantities were compared across tested loci and plotted against within-locus fragment-length heterogeneity, measured as the coefficient of variation of fragment widths (CV).

The DamID-seq background signal was not fully independent of length (Fig. S2A), indicating a weak sample-specific fragment-length bias. However, the observed absolute discrepancy between simple and fragment-length-weighted means substantially exceeded the discrepancy expected from this bias alone. Across the six samples in the Bsh dataset used in this study, the geometric mean ratio of median absolute discrepancies, $\text{median}(D_{obs})/\text{median}(D_{bias})$, was 2.91-fold (95% t-based CI for the population geometric mean: [1.78–4.77]; one-sided paired Wilcoxon signed-rank tests within samples, all $P < 2 \times 10^{-16}$; Fig. S2B, Table S3).

These results indicate that the effect of fragment-length weighting is not primarily explained by systematic fragment-length bias. Instead, the larger observed discrepancy is consistent with the weighted mean better representing spatial occupancy across uneven GATC fragments.

S.2.2 Comparison of differing input normalisation strategies

Lineage-specific factors can exhibit biological asymmetry in binding. One example is Bsh, which in L4 neurons binds at a Notch-dependent open chromatin landscape that is not present in L5 neurons (Xu *et al.*, 2024). In order to understand the impact of different normalisation strategies, a simple zero-preserving scaling approach (via R's internal `scale(center = FALSE, scale = TRUE)`) was compared with cyclic LOESS (the `cyclicloess` method from `limma`) and quantile normalisation. Results were assessed using both subtype-specific scRNA-seq expression markers as a ground truth.

The scaled but otherwise unnormalised dataset showed strong bimodality in the t-statistic distribution, suggesting either unnormalised technical structure, strong biological asymmetry, or both. Quantile normalisation strongly reduced this bimodality, whereas LOESS removed bimodality entirely (Fig S3). However, intersecting the differentially-bound loci from these analyses suggested that LOESS over-corrected the asymmetry of Bsh binding. In L4 neurons, LOESS normalisation appeared to suppress identification of true biological signal, identifying fewer novel binding loci concordant with L4 expression markers (Odds ratio 1.78) than with quantile normalisation (Odds ratio 2.33) (Tables S4, S5). A corollary of this was the identification of over 800 additional L5-enriched loci that lacked any association with L5 subtype markers (Odds ratio 1.12). Conversely, quantile normalisation showed the expected depletion of L5 markers among novel targets (Odds ratio 0.46) (Tables S4, S5).

These results should not be interpreted as a general recommendation of quantile normalisation for all DamID datasets; rather, they illustrate that normalisation choice can affect biological conclusions, and should be carefully assessed against both data diagnostics and known biologi-

cal priors when performing differential analysis. Both cyclic LOESS and quantile normalisation methods on input data are provided by `damidBind`.

S.2.3 Suitability of `NOISeq` for CATaDa analysis

A mean-variance plot of the CATaDa data from Xu *et al.* (2024) shows that these loci-weighted-average count scores are substantially under-dispersed, violating negative binomial assumptions. While `edgeR` can handle fractional count data, and its quasi-likelihood (QL) framework can potentially handle under-dispersion, the non-parametric analysis of `NOISeq` was hypothesised to be a more appropriate fit for these unusual data.

To determine the suitability of `NOISeq` to handle these data, a direct comparison was performed between `NOISeq` and `edgeR` (using `edgeR`'s quasi-likelihood framework) on the lamina neuron CATaDa data used in this study, using both the true biological data and a permuted null test on all nine unique non-biological 3-versus-3 partitions (treating complementary partitions as equivalent and excluding the true L4-versus-L5 split). `NOISeq` identified 3593 loci in the true biological comparison, but only 26.4 loci on average in the mixed-label null contrasts, with a maximum of 70, corresponding to a mean empirical null-call fraction of 0.7%, and a worst-case fraction of 1.9% (Fig. S5A; Table S7). `edgeR` identified 4368 loci in the true biological contrast and no loci in the mixed-label null contrasts (Fig. S5A; Table S7). These results indicate low false-positive behaviour for both methods under permutation with this example dataset. For the true biological condition, almost all (99.6%) of the loci identified by `NOISeq` were also called by `edgeR` (Fig. S5B-E), providing confidence in the `NOISeq` analysis.

For this representative dataset, `NOISeq` thus appears to represent a conservative choice, and does not appear to unduly inflate Type I errors in the permuted null test. While `edgeR` QL also performed well on the example dataset, the underlying data inputs remain as under-dispersed locus-level weighted average intensities rather than raw counts. These analyses support the use of `NOISeq` as a pragmatic, non-parametric solution for analysing locus-averaged CATaDa intensities.

S.2.4 Volcano plot label thinning

Differential analysis typically involves the display of hundreds to thousands of significant loci in volcano plots. Current graphical label packages (e.g. `ggrepel` on thousands of datapoints) within the R ecosystem are limited by total non-overlapping label plot space constraints, meaning that significant point clusters with high density remain unlabelled.

To solve this, `damidBind` uses Algorithm 1 to thin the list of labelled points in dense regions, displaying the thinned list with `ggrepel`. The result is deeper labelling within the significant loci cloud, increasing the number of loci with displayed labels within dense regions of volcano plots, while still preserving labels on isolated points.

S.2.5 Gene occupancy FDR estimation

While `damidBind` is primarily concerned with differential analysis, RNA Polymerase DamID, as a proxy for gene expression, allows an additional option of restricting any downstream visualisation to expressed genes only. The gene body occupancy FDR algorithm used for this purpose by `damidBind` is substantially modified from the original described in Southall *et al.* (2013), using a two-tiered regression modelling approach built upon a permutation-based null distribution. The objective is to create a predictive model (Algorithm 2) that can estimate an empirical p-value for any given gene's observed occupancy score and fragment count (Algorithm 3). The per-gene p-values from each replicate are grouped by test condition during the

Algorithm 1: Sampling points by local isolation

Input : D: A dataframe containing coordinates
k: An integer for both search depth and priority calculation
r: A fixed numeric exclusion radius
Output : V_{kept}: A logical vector indicating points selected for labelling

`# Pre-process, calculate isolation priority and sort`
$X \leftarrow$ Extract (x, y) coordinates from D
$X_{scaled} \leftarrow$ Standardise X (centre and scale to unit variance)
$N_{dist} \leftarrow$ For each point in X_{scaled}, find distances to its k nearest neighbours
$P \leftarrow$ Calculate the median of N_{dist} for each point $S \leftarrow$ Indices of points sorted by P in descending order

`# Iterative thinning via radius-based exclusion`
Initialise $V_{processed}$ as a boolean vector of size $|D|$ set to *False*
Initialise V_{kept} as a boolean vector of size $|D|$ set to *False*
foreach *index* $i \in S$ **do**
    if $V_{processed}[i]$ *is False* **then**
        $V_{kept}[i] \leftarrow$ *True*
        $V_{processed}[i] \leftarrow$ *True*
        $R_{idx} \leftarrow$ Indices of all points in X_{scaled} within distance r of $X_{scaled}[i]$
        foreach *index* $j \in R_{idx}$ **do**
            $V_{processed}[j] \leftarrow$ *True*
        end
    end
end
return V_{kept}

`differential_binding()` call and aggregated using either Fisher's (Fisher, 1925) or Stouffer's (Stouffer *et al.*, 1949) method, to give a per-condition p-value for each gene. Finally, these are adjusted for multiple hypothesis testing using the Benjamini-Hochberg method (Benjamini and Hochberg, 1995) to yield a final per-gene occupancy FDR.

When applied to correctly-normalised RNA Polymerase DamID data, the occupancy FDR serves as a proxy for gene expression (Southall *et al.*, 2013), although genes with significant paused RNA Polymerase may also be detected using this approach. The gene occupancy FDR values are not directly used to determine differential gene expression changes, but are used to filter the downstream plots and analysis for expressed genes only.

The major algorithmic changes from Southall *et al.* (2013) in the method implemented in `damidBind` include:

1. The use of fragment-length-weighted mean values for building the null distribution.

2. Generating the underlying null model distributions by sampling with replacement.

3. The use of log-linear models for the Tier 1 regressions.

4. Using natural spline fits for the Tier 2 regressions (slope, intercept, mean squared error (MSE) of residuals) to capture non-linear relationships across the range of fragment counts.

5. The use of weighted least squares (WLS) for Tier 2 slope and intercept models, using the

inverse of the Tier 1 standard errors as weights, to account for model heteroscedasticity (Baskerville, 1972).

6. Applying a bias correction factor derived from the predicted MSE when back-transforming from the log-scale, to correct for Jensen's inequality (Finney, 1941; Baskerville, 1972).

7. Determining a condition-level FDR by combining unadjusted replicate p-values using either Stouffer's Z-score method or Fisher's method, followed by Benjamini-Hochberg (BH) adjustment at the condition level.

In order to capture the non-linear relationship between fragment counts and the regression parameters for the Tier 2 regressions, the slope and intercept are modelled as a function of the log-transformed fragment count (Fig. S8A, B). The pseudo-linear residual variance (MSE) is modelled on the linear fragment count (Fig. S8C) to accurately represent the variance profile in low fragment count genes.

Algorithm 2: Occupancy FDR: Model training

Input : S_{genome}: Binding profile scores
W_{genome}: Fragment widths
N_{iter}: Simulation iterations
C_{frag}: Set of fragment counts
T_{occ}: Set of occupancy thresholds
Output : Models M_{slope}, M_{int}, and M_{MSE}

`# Simulate null distribution by sampling fragments`
Initialise matrix H of size $|T_{\text{occ}}| \times |C_{\text{frag}}|$ with zeros
for $n \leftarrow 1$ **to** N_{iter} **do**
  foreach $f \in C_{\text{frag}}$ **do**
    Idx $\leftarrow$ Sample f indices from profile with replacement
    $\text{occ}_{\text{rand}} \leftarrow \frac{\sum_{i \in \text{Idx}} S_{\text{genome}}[i] \cdot W_{\text{genome}}[i]}{\sum_{i \in \text{Idx}} W_{\text{genome}}[i]}$ `# Weighted mean of GATC fragments`
    foreach $t \in T_{\text{occ}}$ **do**
      if $\text{occ}_{\text{rand}} > t$ **then**
        $H[t, f] \leftarrow H[t, f] + 1$
      end
    end
  end
end
$P \leftarrow H/N_{\text{iter}}$ `# Empirical probabilities`

`# Tier 1: Log-linear regression for each fragment count`
foreach $f \in C_{\text{frag}}$ **do**
  $P_f \leftarrow$ Probability column for f; $T_f \leftarrow T_{\text{occ}}$
  Remove indices where $P_f = 0$
  if remaining indices ≥ 3 **then**
    Fit M_f: $\log(P_f) \sim T_f$
    Extract coefficients ($\beta_{\text{slope},f}$, $\beta_{\text{int},f}$), standard errors ($\text{SE}_{\text{slope},f}$, $\text{SE}_{\text{int},f}$), and residual variance (MSE_f)
  end
end

`# Tier 2: Weighted natural spline (NS) regressions`
$f_{\log} \leftarrow \log(f)$
Fit M_{slope}: $\beta_{\text{slope}} \sim \text{NS}(f_{\log}, \text{df} = 3)$ with weights $1/(\text{SE}_{\text{slope}} + \epsilon)$
Fit M_{int}: $\beta_{\text{int}} \sim \text{NS}(f_{\log}, \text{df} = 3)$ with weights $1/(\text{SE}_{\text{int}} + \epsilon)$
Fit M_{MSE}: $\text{MSE} \sim \text{NS}(f, \text{df} = 3)$ `# Unweighted`
return $M_{\text{slope}}, M_{\text{int}}, M_{\text{MSE}}$

Algorithm 3: Occupancy FDR: p-value estimates and replicate integration

Input : G: Set of genes with observed occupancy and fragment counts per replicate
C: Set of test conditions linked to replicates r
$M_{\text{slope},r}, M_{\text{int},r}, M_{\text{MSE},r}$: Pre-trained Tier 2 models for each replicate r
Method: Statistical approach (Stouffer or Fisher)
Output : G with condition-level adjusted g.FDR$_c$ values

```
# Estimate individual replicate p-values
foreach condition $c \in C$ do
    foreach replicate $r \in c$ do
        foreach gene $g \in G$ do
            if $g$.nfrag $> 0$ then
                $\text{pred}_{\text{slope}} \leftarrow$ Predict from $M_{\text{slope},r}$ using $\log(g.\text{nfrag})$
                $\text{pred}_{\text{int}} \leftarrow$ Predict from $M_{\text{int},r}$ using $\log(g.\text{nfrag})$
                $\text{pred}_{\text{MSE}} \leftarrow \max(0,$ Predict from $M_{\text{MSE},r}$ using $g.\text{nfrag})$
                $\log(p) \leftarrow (\text{pred}_{\text{slope}} \times g.\text{occ}) + \text{pred}_{\text{int}}$
                $p_{g,r} \leftarrow \exp(\log(p) + \text{pred}_{\text{MSE}}/2)$        # Jensen's correction
                $p_{g,r} \leftarrow \text{clamp}(p_{g,r}, \text{min} = 10^{-16}, \text{max} = 1.0)$
            end
            else
                $p_{g,r} \leftarrow 1.0$
            end
        end
    end

    # Aggregate p-values across replicates per condition
    foreach gene $g \in G$ do
        $k \leftarrow$ Count of replicates for $g$ where $p_{g,r} < 1.0$
        if $k = 1$ then
            $P_{\text{combined},g} \leftarrow$ the single non-1.0 p-value
        end
        else if Method is Stouffer then
            foreach $r \in c$ do
                $z_r \leftarrow \Phi^{-1}(1 - p_{g,r})$        # Inverse Normal CDF
            end
            $Z_{\text{meta}} \leftarrow \frac{\sum z_r}{\sqrt{k}}$        # Unweighted combination
            $P_{\text{combined},g} \leftarrow 1 - \Phi(Z_{\text{meta}})$        # Back-transform
        end
        else if Method is Fisher then
            $X^2 \leftarrow -2 \sum_{r=1}^{k} \log(p_{g,r})$        # Fisher statistic
            $P_{\text{combined},g} \leftarrow P(\chi^2_{2k} > X^2)$        # Chi-squared lookup
        end
    end

    # FDR correction at condition level
    $G.\text{FDR}_c \leftarrow$ Benjamini-Hochberg$(P_{\text{combined}})$
    return $G$ with assigned $G.\text{FDR}_c$
end
```

Supplementary figures

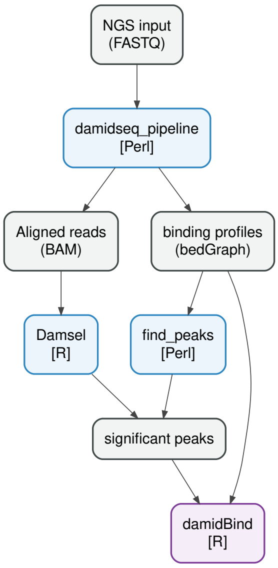

Figure S1: Suggested workflow options for DamID-seq data processing, from raw sequencing reads to `damidBind`.

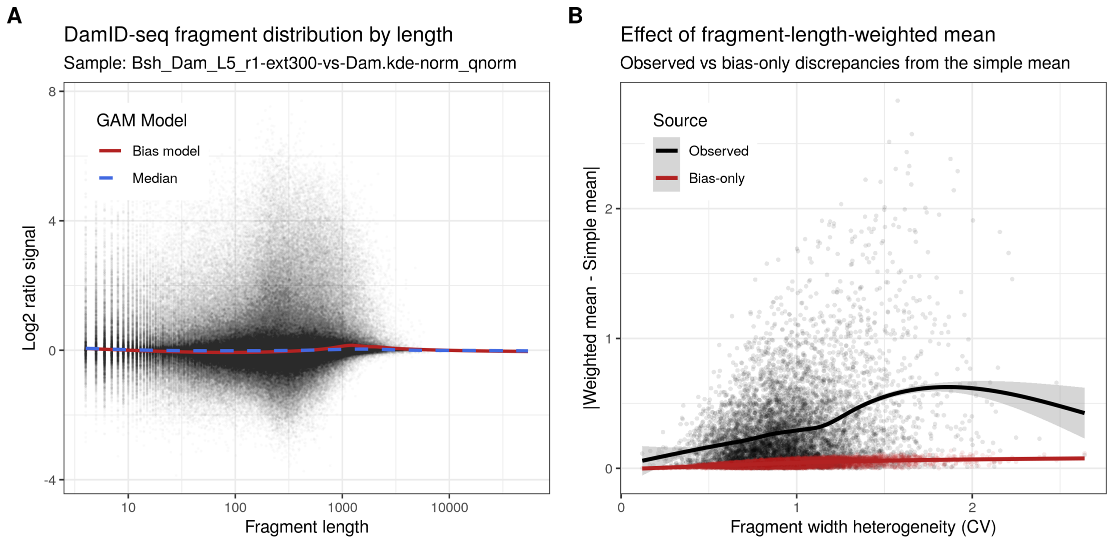

Figure S2: Discrepancies between fragment-length-weighted averaging and simple averaging are not driven by systematic bias. (A) Plots of the underlying fragment length bias over all non-peak GATC fragments, used to fit a null model of systematic fragment-length signal bias (red); a fit of the signal median is also shown. (B) Discrepancy between simple and fragment-length-weighted means, both from the data (Observed, black) and as predicted using the bias-only model (Bias-only, red).

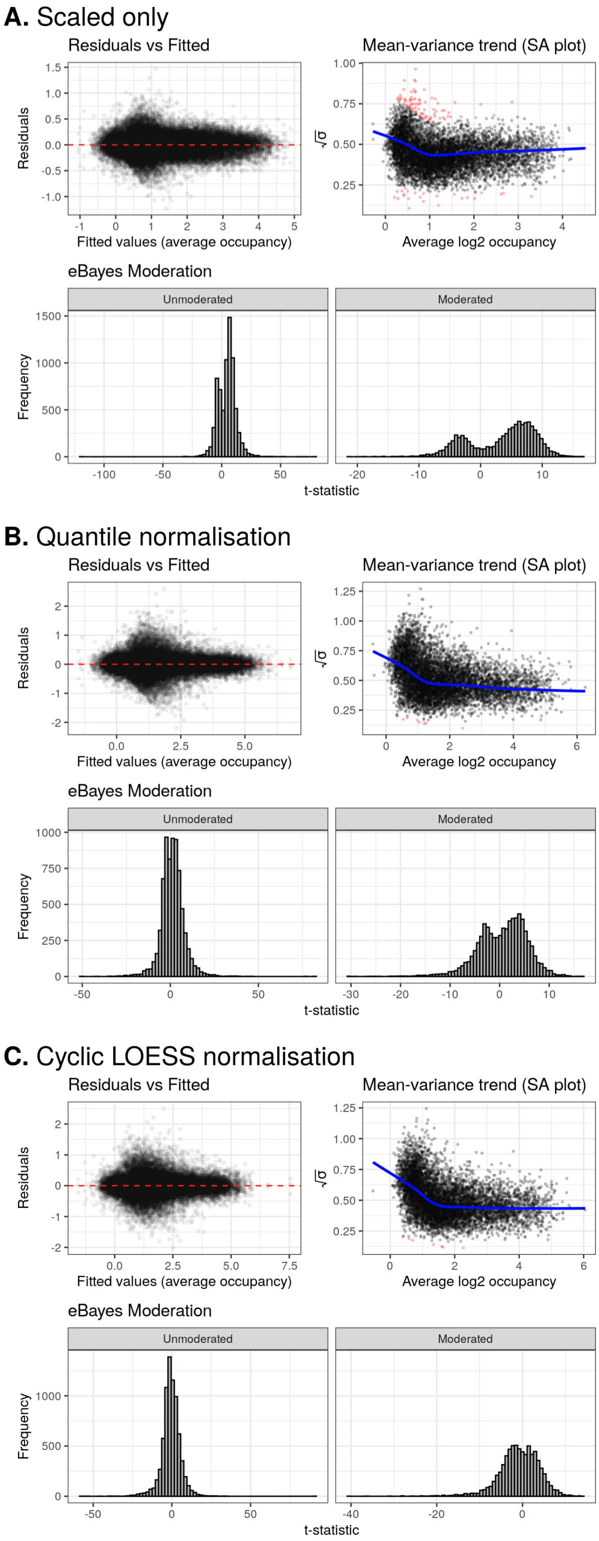

Figure S3: Diagnostic plots for `limma`'s eBayes moderation generated by `damidBind` using Bsh L4/L5 binding dataset with only zero-preserving scaling, with quantile normalisation, and with cyclic LOESS normalisation. Plots showing the heteroscedasticity of the data, the SA plot showing the model fit to the mean-variance of the data (outliers coloured red; fit line in blue), and the effect of eBayes moderation on t-statistic shrinkage.

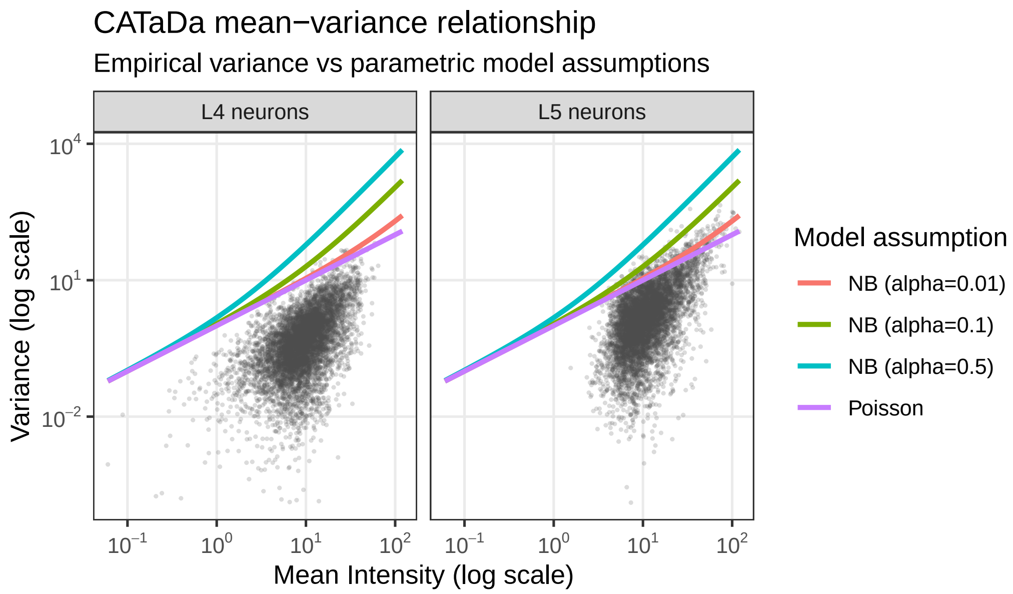

Figure S4: Plots of mean vs variance relationship of CATaDa peak data. Data for L4 and L5 neuronal subtypes (from (Xu *et al.*, 2024)) are shown, alongside Poisson (Var $= \mu$) and Negative Binomial (Var $= \mu + \alpha\mu^2$) responses; points represent individual loci. Input data was loaded with `pre_scale=FALSE`, `norm_method="none"` (i.e. no normalisation was applied to the dataset). In both cases, the data sit below the model lines and are under-dispersed, violating NB and Poisson assumptions.

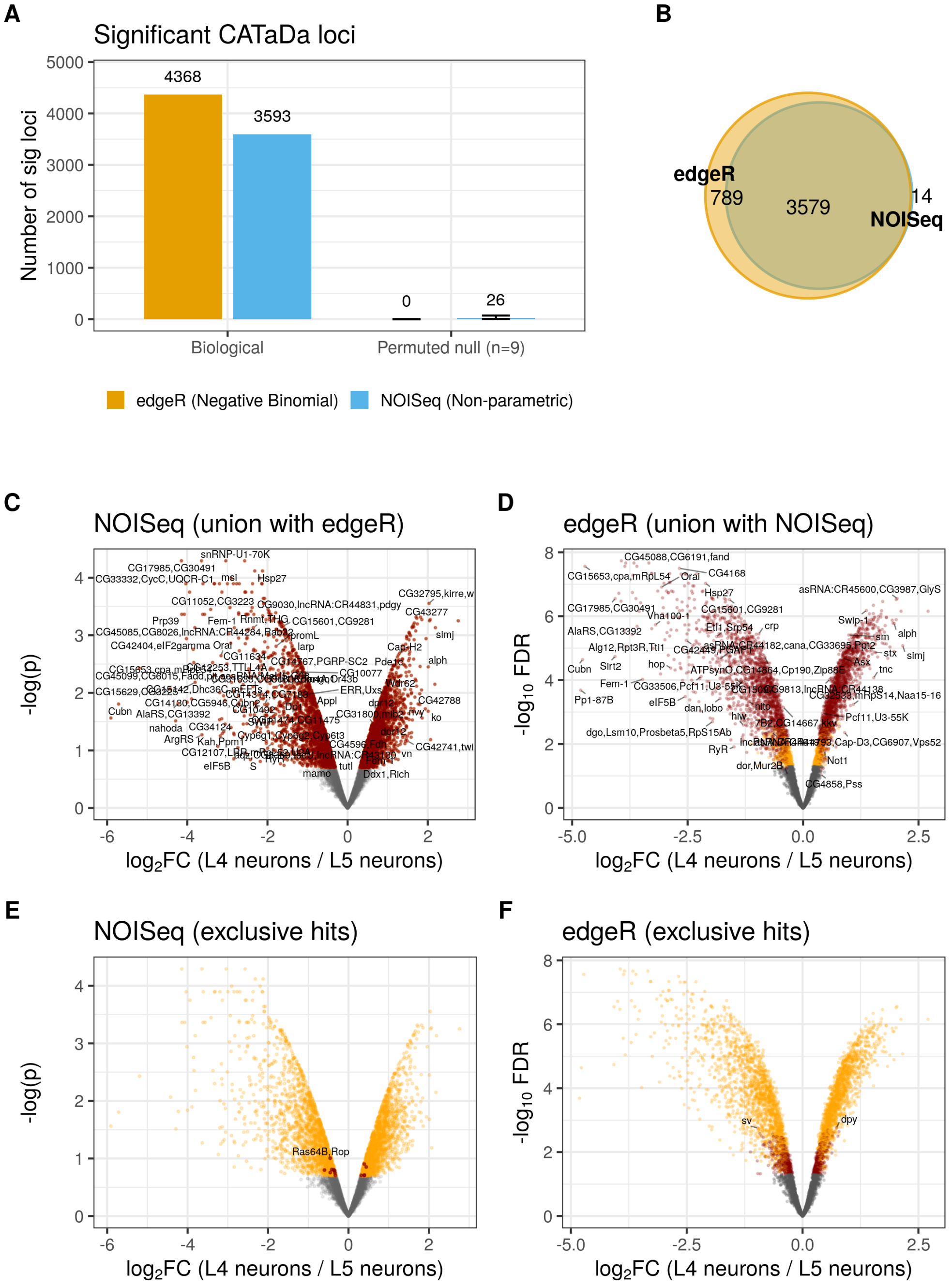

Figure S5: Comparison of `NOISeq` and `edgeR` performance on CATaDa data. (A) Significant hits generated by both methods, for both the biological lamina neuron data from Xu *et al.* (2024) and a permuted null dataset which should yield no hits, to assess FDR performance. (B) Venn diagram of overlapping significant loci called by the two methods on the lamina dataset. (C and D) Significant `NOISeq` and `edgeR` loci, coloured by set overlap in red, other significant loci in orange. (E and F) Significant `NOISeq` and `edgeR` loci, coloured by set uniqueness in red, other significant loci in orange.

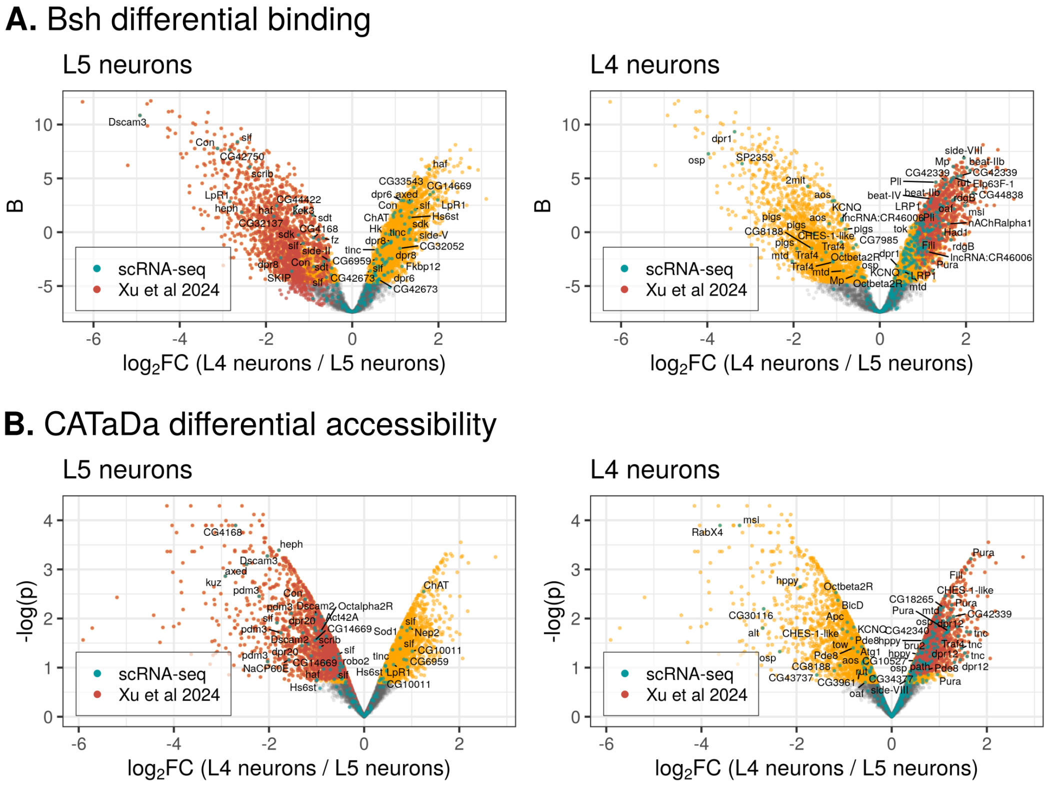

Figure S6: Comparison of `damidBind`'s performance against previously published analysis from Xu *et al.* (2024). (A) Bsh TF differential binding; (B) CATaDa differential accessibility. In all plots, significant loci identified by `damidBind` are coloured orange; significant loci identified by Xu *et al.* (2024) are coloured red; scRNA-seq gene expression markers uniquely expressed in each lineage (data from (Xu *et al.*, 2024)) are coloured cyan. See also Table S4. Results using quantile normalisation are shown.

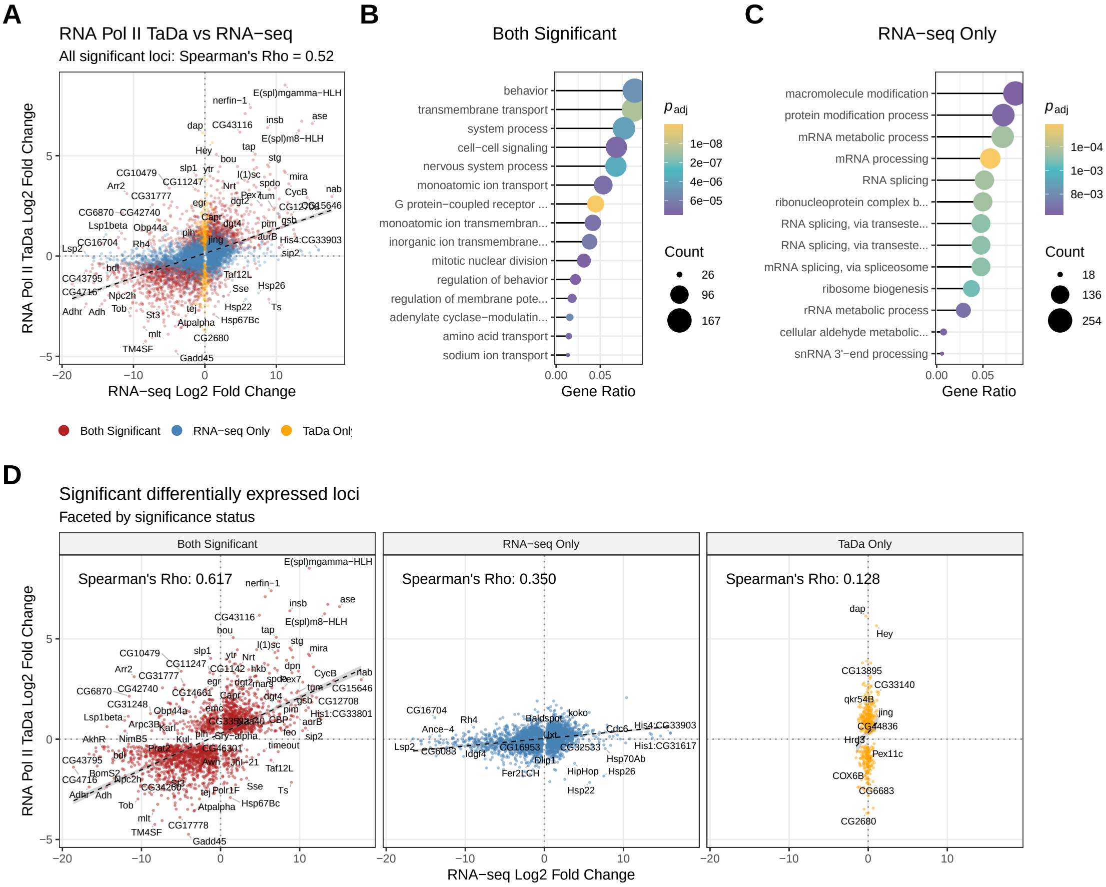

Figure S7: Differential expression compared between TaDa and RNA-seq. In both cases, third instar larval neural stem cells were compared to adult neurons. (A) All significant loci compared; (B) faceted by significance status; (C) Gene Ontology (GO) enrichment terms by significance class (no GO terms were significantly enriched for the TaDa-only significance group). See Supplementary Methods for details.

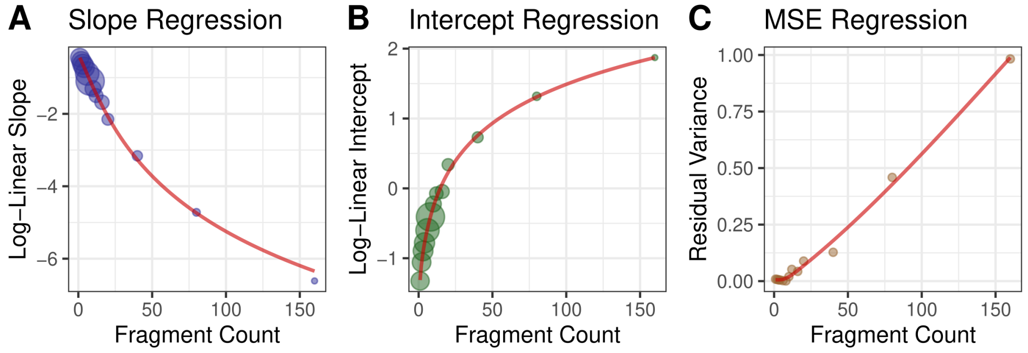

Figure S8: FDR modelling for RNA Polymerase occupancy data. The plots show the Tier 2 regression fits and Tier 1 model heteroscedasticity, for a typical DamID RNA Polymerase dataset. Both the Tier 2 slope (A) and intercept (B) regressions use weighted natural spline fits to fit the underlying data; circle size is proportional to weight. The model mean squared error (MSE) increases with fragment count (C) and is also fitted with an unweighted natural spline to apply the appropriate model bias correction upon back-transformation. Data illustrated is derived from RNA Polymerase (RpII215) occupancy data from GSE77860.

Supplementary tables

Table S1: Summary of DamID-seq-related bioinformatics software

**Package**	**Function**	**Outputs**
damidseq_pipeline	NGS sequencing alignment, Dam-fusion/Dam-only signal normalisation, and $\log_2$ ratio generation, or RPM occupancy counts in the case of CATaDa.	bedGraph profiles and BAM alignments
Damsel	Identifies significant peaks from individual replicates of Dam-fusion/Dam-only DamID-seq data, using BAM inputs.	dm_results object; peaks(dm_results) provides the peaks as a GenomicRanges object
find_peaks	Per-replicate peak caller on $\log_2$ ratio datasets. Finds contiguous high-signal regions that pass an FDR threshold.	BED or GFF peak files
damidBind	Performs differential binding, expression, or accessibility analysis on input profiles generated by damidseq_pipeline, along with peaks generated via Damsel or find_peaks.	DamIDResults object

Table S2: Benchmark results for damidBind workflows using serial and multicore execution. Significantly shorter execution times result from parallel processing, although the benefits of using more than 15 cores is limited.

Workflow	Execution backend[a]	Median	Min	Total time	n_{itr}
load_data_genes()	serial	15.11 min	15.08 min	1.26 h	5
load_data_genes()	multicore_15	1.80 min	1.78 min	9.23 min	5
load_data_genes()	multicore_30	1.70 min	1.67 min	8.47 min	5
load_data_peaks()	serial	1.16 min	1.15 min	5.82 min	5
load_data_peaks()	multicore_15	13.99 s	13.21 s	1.15 min	5
load_data_peaks()	multicore_30	12.06 s	11.89 s	1.06 min	5

[a] Execution backend corresponds to: BiocParallel::SerialParam(), BiocParallel::MulticoreParam(workers = 15), and BiocParallel::MulticoreParam(workers = 30) respectively.

Table S3: Assessment of fragment-length weighted averaging, Observed vs. Bias-only.

Sample	Median D_{obs}[a]	Median D_{bias}[b]	Ratio	p-value[c]	CI lower	CI upper
Bsh_Dam_L4_r1	0.153	0.0840	1.82	$< 2 \times 10^{-16}$	1.75	1.89
Bsh_Dam_L4_r2	0.152	0.0790	1.93	$< 2 \times 10^{-16}$	1.82	2.04
Bsh_Dam_L4_r3	0.155	0.0787	1.98	$< 2 \times 10^{-16}$	1.91	2.06
Bsh_Dam_L5_r1	0.196	0.0383	5.13	$< 2 \times 10^{-16}$	4.96	5.31
Bsh_Dam_L5_r2	0.189	0.0457	4.13	$< 2 \times 10^{-16}$	3.98	4.26
Bsh_Dam_L5_r3	0.197	0.0477	4.12	$< 2 \times 10^{-16}$	4.02	4.26

[a] Discrepancy $\bar{S}_{weighted} - \bar{S}_{simple}$
[b] Determined from GAM fit of `non-peak_signal ~ s(log10(fragment_length))`
[c] Wilcoxon signed-rank test

Table S4: Bsh differential binding overlap with ground-truth scRNA-seq (quantile normalisation)

Comparison	Detected	Overlap[a]	Overlap (%)	Odds ratio[b]	p-value[b]
L4 (Package total)	2804	191	6.81	2.50	2.65×10^{-15}
L4 (Xu et al.)	856	67	7.83	2.09	1.17×10^{-6}
L4 (Novel only)	1948	124	6.37	2.33	1.53×10^{-10}
L5 (Package total)	1869	27	1.44	0.56	5.00×10^{-3}
L5 (Xu et al.)	1551	23	1.48	0.59	2.04×10^{-2}
L5 (Novel only)	419	5	1.19	0.46	9.95×10^{-2}

[a] Overlap with subtype-specific gene expression determined from scRNA-seq.
[b] Fisher's exact test.

Table S5: Overlap with ground-truth scRNA-seq (LOESS normalisation)

Comparison	Detected	Overlap[a]	Overlap (%)	Odds ratio[b]	p-value[b]
L4 (Package total)	1694	117	6.91	2.01	1.69×10^{-8}
L4 (Xu et al.)	856	67	7.83	2.11	9.74×10^{-7}
L4 (Novel only)	866	53	6.12	1.79	5.86×10^{-4}
L5 (Package total)	2295	44	1.92	0.80	2.34×10^{-1}
L5 (Xu et al.)	1568	23	1.47	0.59	2.06×10^{-2}
L5 (Novel only)	815	22	2.70	1.12	6.25×10^{-1}

[a] Overlap with subtype-specific gene expression determined from scRNA-seq.
[b] Fisher's exact test.

Table S6: Recovery rates of original loci from Xu et al. across normalisation conditions

Condition	Total (Xu et al.)	Recovered	% Recovery
L4 quantile norm (locus)	856	856	100.00
L4 quantile norm (gene)	1271	1271	100.00
L5 quantile norm (locus)	1584	1450	91.54
L5 quantile norm (gene)	1451	1374	94.69
L4 loess norm (locus)	856	828	96.73
L4 loess norm (gene)	1271	1242	97.72
L5 loess norm (locus)	1584	1480	93.43
L5 loess norm (gene)	1461	1405	96.17

Table S7: CATaDa permuted null performance by method.

Method	Biological calls	Mean null calls	Maximum null calls	Mean empirical null-call fraction	Maximum empirical null-call fraction
`NOISeq` (q=0.8)	3593	26.4	70	0.7%	1.9%
`edgeR` (QL)	4368	0.0	0	0.0%	0.0%

Table S8: CATaDa differential accessibility overlap with ground-truth scRNA-seq (quantile normalisation)

Comparison	Detected	Overlap[a]	Overlap (%)	Odds ratio[b]	p-value[b]
L4 (Package total)	1731	114	6.59	2.29	3.42×10^{-10}
L4 (Xu et al.)	1356	92	6.78	2.20	1.64×10^{-8}
L4 (Novel only)	375	22	5.87	2.02	5.30×10^{-3}
L5 (Package total)	1862	48	2.58	1.56	2.13×10^{-2}
L5 (Xu et al.)	1724	45	2.61	1.57	1.86×10^{-2}
L5 (Novel only)	159	4	2.52	1.54	3.42×10^{-1}

[a] Overlap with subtype-specific gene expression determined from scRNA-seq.
[b] Fisher's exact test.

Table S9: Recovery rates of original CATaDa results from Xu et al.

Condition	Total (Xu et al.)	Recovered	% Recovery
L4 (locus)	1356	1356	100.00
L4 (gene)	1886	1886	100.00
L5 (locus)	1724	1703	98.78
L5 (gene)	2889	2861	99.03

Table S10: Directional concordance between RNA Polymerase TaDa and bulk RNA-seq differential analysis

Gene set considered	Concordant	Total	Directional concordance (%)
Significant in either assay	4330	6058	71.5
Significant in TaDa	2116	2631	80.4
Significant in RNA-seq	4086	5633	72.5
Significant in TaDa, $\lvert \log_2 \mathrm{FC} \rvert > 2$	251	298	84.2
Significant in RNA-seq, $\lvert \log_2 \mathrm{FC} \rvert > 2$	2303	2817	81.8

Concordance was defined as the same direction of $\log_2$ fold-change in RNA Polymerase TaDa and RNA-seq. "Significant in either assay" refers to genes significant in at least one of the two analyses.

Supplementary references

Aughey, G.N, Estacio Gomez, A, Thomson, J, Yin, H and Southall, T.D (2018). CATaDa Reveals Global Remodelling of Chromatin Accessibility during Stem Cell Differentiation in Vivo. *eLife* **7**, 1–22.

Baskerville, G.L (1972). Use of Logarithmic Regression in the Estimation of Plant Biomass. *Can. J. For. Res.* **2**, 49–53.

Benjamini, Y and Hochberg, Y (1995). Controlling the False Discovery Rate: A Practical and Powerful Approach to Multiple Testing. *Royal Statistical Society. Journal. Series B: Methodological* **57**, 289–300.

Berger, C, Harzer, H, Burkard, T.R, Steinmann, J, van der Horst, S, Laurenson, A.S, Novatchkova, M, Reichert, H and Knoblich, J.A (2012). FACS Purification and Transcriptome Analysis of Drosophila Neural Stem Cells Reveals a Role for Klumpfuss in Self-Renewal. *Cell Rep.* **2**, 407–418.

Cheetham, S.W, Gruhn, W.H, van den Ameele, J, Krautz, R, Southall, T.D, Kobayashi, T, Surani, M.A and Brand, A.H (2018). Targeted DamID Reveals Differential Binding of Mammalian Pluripotency Factors. *Development* **145**, dev170209.

Chen, Y, Chen, L, Lun, A.T.L, Baldoni, P.L and Smyth, G.K (2025). edgeR v4: Powerful Differential Analysis of Sequencing Data with Expanded Functionality and Improved Support for Small Counts and Larger Datasets. *Nucleic Acids Res* **53**.

Doupé, D.P, Marshall, O.J, Dayton, H, Brand, A.H and Perrimon, N (2018). Drosophila Intestinal Stem and Progenitor Cells Are Major Sources and Regulators of Homeostatic Niche Signals. *Proc. Natl. Acad. Sci.* **115**, 12218–12223.

Finney, D.J (1941). On the Distribution of a Variate Whose Logarithm Is Normally Distributed. *Suppl. J. R. Stat. Soc.* **7**, 155–161.

Fisher, R (1925). *Statistical Methods for Research Workers, 11th Ed. Rev* Edinburgh, Oliver and Boyd.

Gentleman, R.C, Carey, V.J, Bates, D.M, Bolstad, B, Dettling, M, Dudoit, S, Ellis, B, Gautier, L, Ge, Y, Gentry, J, Hornik, K, Hothorn, T, Huber, W, Iacus, S, Irizarry, R, Leisch, F, Li, C, Maechler, M, Rossini, A.J, Sawitzki, G, Smith, C, Smyth, G, Tierney, L, Yang, J.Y and Zhang, J (2004). Bioconductor: Open Software Development for Computational Biology and Bioinformatics. *Genome Biol* **5**, R80.

Gervais, L, van den Beek, M, Josserand, M, Sallé, J, Stefanutti, M, Perdigoto, C.N, Skorski, P, Mazouni, K, Marshall, O.J, Brand, A.H, Schweisguth, F and Bardin, A.J (2019). Stem Cell Proliferation Is Kept in Check by the Chromatin Regulators Kismet/CHD7/CHD8 and Trr/MLL3/4. *Dev. Cell* **49**, 556–573.

Hulsen, T, de Vlieg, J and Alkema, W (2008). BioVenn - a Web Application for the Comparison and Visualization of Biological Lists Using Area-Proportional Venn Diagrams. *BMC genomics* **9**, 488.

Lawrence, M, Huber, W, Pagès, H, Aboyoun, P, Carlson, M, Gentleman, R, Morgan, M.T and Carey, V.J (2013). Software for Computing and Annotating Genomic Ranges. *PLoS Comput Biol* **9**, e1003118.

Love, M.I, Huber, W and Anders, S (2014). Moderated Estimation of Fold Change and Dispersion for RNA-seq Data with DESeq2. *Genome Biol* **15**, 550.

Marshall, O.J and Brand, A.H (2015). Damidseq_pipeline: An Automated Pipeline for Processing DamID Sequencing Datasets. *Bioinformatics* **31**, 3371–3.

Marshall, O.J and Brand, A.H (2017). Chromatin State Changes during Neural Development Revealed by in Vivo Cell-Type Specific Profiling. *Nat. Commun.* **8**, 2271.

Marshall, O.J, Southall, T.D, Cheetham, S.W and Brand, A.H (2016). Cell-Type-Specific Pro-

filing of Protein–DNA Interactions without Cell Isolation Using Targeted DamID with next-Generation Sequencing. *Nat. Protoc.* **11**, 1586–1598.

Nguyen, P.K, Froldi, F, McMullen, J.P.D, Southall, T.D, Marshall, O.J and Cheng, L.Y (2026). Chinmo Defines the Region-Specific Oncogenic Competence in the Drosophila Central Nervous System. *Proc. Natl. Acad. Sci.* **123**, e2534053123.

Otsuki, L and Brand, A.H (2018). Cell Cycle Heterogeneity Directs the Timing of Neural Stem Cell Activation from Quiescence. *Science* **360**, 99–102.

Page, C.G, Lonsdale, A, Mitchell, K.A, Schröder, J, Harvey, K.F and Oshlack, A (2024). Damsel: Analysis and Visualisation of DamID Sequencing in R. *Bioinformatics* **40**, btae695.

Perlegos, A.E, Byrns, C.N and Bonini, N.M (2024). Cell Type-Specific Regulation of m6A Modified RNAs in the Aging Drosophila Brain. *Aging Cell* **23**, e14076.

R_Development_Core_Team (2011). R: A Language and Environment for Statistical Computing.

Smyth, G.K (2005). Limma: Linear Models for Microarray Data. In Gentleman, R, Carey, V.J, Huber, W, Irizarry, R.A and Dudoit, S (eds), *Bioinformatics and Computational Biology Solutions Using R and Bioconductor* Springer, New York, NY , pp. 397–420.

Southall, T.D, Gold, K.S, Egger, B, Davidson, C.M, Caygill, E.E, Marshall, O.J and Brand, A.H (2013). Cell-Type-Specific Profiling of Gene Expression and Chromatin Binding without Cell Isolation: Assaying RNA Pol II Occupancy in Neural Stem Cells. *Dev. Cell* **26**, 101–12.

Stouffer, S.A, Suchman, E.A, Devinney, L.C, Star, S.A and Williams Jr., R.M (1949). *The American Soldier: Adjustment during Army Life. (Studies in Social Psychology in World War II), Vol. 1* Princeton Univ. Press, Oxford, England.

Tarazona, S, Furió-Tarí, P, Turrà, D, Di Pietro, A, Nueda, M.J, Ferrer, A and Conesa, A (2015). Data Quality Aware Analysis of Differential Expression in RNA-seq with NOISeq R/Bioc Package. *Nucleic Acids Res.* **43**.

Tosti, L, Ashmore, J, Tan, B.S.N, Carbone, B, Mistri, T.K, Wilson, V, Tomlinson, S.R and Kaji, K (2018). Mapping Transcription Factor Occupancy Using Minimal Numbers of Cells in Vitro and in Vivo. *Genome Res.* **28**, 592–605.

van Steensel, B and Henikoff, S (2000). Identification of in Vivo DNA Targets of Chromatin Proteins Using Tethered Dam Methyltransferase. *Nat. Biotechnol.* **18**, 424–8.

van Steensel, B, Delrow, J and Henikoff, S (2001). Chromatin Profiling Using Targeted DNA Adenine Methyltransferase. *Nat. Genet.* **27**, 304–8.

Xu, C, Ramos, T.B, Marshall, O.J and Doe, C.Q (2024). Notch Signaling and Bsh Homeodomain Activity Are Integrated to Diversify Drosophila Lamina Neuron Types. *eLife* **12**, RP90136.

Yu, G, Wang, L.G, Han, Y and He, Q.Y (2012). ClusterProfiler: An R Package for Comparing Biological Themes among Gene Clusters. *OMICS J. Integr. Biol.* **16**, 284–287.

Zhu, A, Ibrahim, J.G and Love, M.I (2019). Heavy-Tailed Prior Distributions for Sequence Count Data: Removing the Noise and Preserving Large Differences. *Bioinformatics* **35**, 2084–2092.
